# Social Media Usage for Medical Education and Smartphone Addiction Among Medical Students: National Web-Based Survey

**DOI:** 10.2196/55149

**Published:** 2024-10-22

**Authors:** Thomas Clavier, Emma Chevalier, Zoé Demailly, Benoit Veber, Imad-Abdelkader Messaadi, Benjamin Popoff

**Affiliations:** 1 Department of Anesthesiology, Critical Care and Perioperative Medicine, CHU Rouen, 1 rue de Germont, Rouen, 76031, France, 33 232888990; 2 INSERM U1096, Normandie Universite, UNIROUEN, Rouen, France; 3 Rouen Faculty of Medicine, Normandie Universite, UNIROUEN, Rouen, France

**Keywords:** medical student, social network, social media, smartphone addiction, medical education, mobile addiction, social networks

## Abstract

**Background:**

Social media (SoMe) have taken a major place in the medical field, and younger generations are increasingly using them as their primary source to find information.

**Objective:**

This study aimed to describe the use of SoMe for medical education among French medical students and assess the prevalence of smartphone addiction in this population.

**Methods:**

A cross-sectional web-based survey was conducted among French medical students (second to sixth year of study). The questionnaire collected information on SoMe use for medical education and professional behavior. Smartphone addiction was assessed using the Smartphone Addiction Scale Short-Version (SAS-SV) score.

**Results:**

A total of 762 medical students responded to the survey. Of these, 762 (100%) were SoMe users, spending a median of 120 (IQR 60‐150) minutes per day on SoMe; 656 (86.1%) used SoMe for medical education, with YouTube, Instagram, and Facebook being the most popular platforms. The misuse of SoMe in a professional context was also identified; 27.2% (207/762) of students posted hospital internship content, and 10.8% (82/762) searched for a patient’s name on SoMe. Smartphone addiction was prevalent among 29.1% (222/762) of respondents, with a significant correlation between increased SoMe use and SAS-SV score (*r*=0.39, 95% CI 0.33‐0.45; *P*<.001). Smartphone-addicted students reported a higher impact on study time (211/222, 95% vs 344/540, 63.6%; *P*<.001) and a greater tendency to share hospital internship content on social networks (78/222, 35.1% vs 129/540, 23.8%; *P*=.002).

**Conclusions:**

Our findings reveal the extensive use of SoMe for medical education among French medical students, alongside a notable prevalence of smartphone addiction. These results highlight the need for medical schools and educators to address the responsible use of SoMe and develop strategies to mitigate the risks associated with excessive use and addiction.

## Introduction

In today’s globalized world, social media (SoMe) have taken a significant role in the medical field, serving as essential tools for promoting research, medical innovations, and updates from various specialties (eg, techniques and congresses). With the explosion in the number of platforms and their evolution, several definitions of SoMe have been proposed and gradually amended [1,2]. Recent definitions agree that true SoMe are defined as “web-based technologies that facilitate multi-user interaction that goes beyond fact sharing, centering around content creation, curation, and community engagement, placing user interaction at their art” [3]. These platforms include, for example, major actors like Facebook, Twitter (now X), Instagram, YouTube, TikTok, Snapchat, LinkedIn, or WhatsApp, and exclude websites or blogs with comment sections and podcasts due to their primarily unidirectional nature [3]. SoMe are now widely used by health care professionals for numerous purposes, such as education, patient communication, and colleague discussions [4,5]. SoMe provide a platform for the rapid dissemination of research findings and facilitate networking and collaboration among researchers and clinicians worldwide [6].

Younger generations increasingly rely on SoMe as their primary source of information about brands or organizations, with this usage even surpassing that of internet search engines among 16‐ to 24-year-olds [7]. The main reason for using SoMe is to “stay up-to-date with news and current events” [8]. Time spent on SoMe has consistently grown, rising from 1 hour and 51 minutes per day in 2015 to 2 hours and 24 minutes per day in 2023 [8]. Furthermore, several studies have documented the benefits of using SoMe for medical education [9,10]. Consequently, a growing number of educators and medical societies are leveraging SoMe to showcase their educational content [11,12]. The COVID-19 pandemic has acted as an amplifier of the trend toward distance learning, with SoMe playing a significant role in this regard [13,14]. However, there is a lack of comprehensive data on medical students’ use of these educational resources from SoMe for knowledge acquisition.

Several studies have identified significant risks associated with prolonged SoMe use. Notably, smartphone addiction correlates with the intensity of SoMe usage [15]. This addiction, in turn, can negatively impact students’ quality of life, leading to sleep disorders, musculoskeletal disorders, severe social withdrawal, decreased physical activity, and hypertension [16-19]. Finally, all available data on smartphone addiction among medical students originate from Asia, with no data from Western countries.

The purpose of this study was to address the gap in knowledge regarding the use of SoMe by medical students in Western countries, specifically in France, for medical education. We aimed to describe, on a nationwide scale, how medical students use SoMe for medical learning, their motivations and preferences, and the extent to which they rely on these platforms for educational purposes. Additionally, we sought to determine the prevalence of smartphone addiction in this population and explore its potential impact on academic performance and professional behavior. We hypothesized that a significant proportion of medical students use SoMe for educational purposes; that this usage correlates with specific patterns of SoMe behavior, including misuse such as breaches of patient confidentiality; and that high levels of SoMe use are associated with increased rates of smartphone addiction.

## Methods

### Objectives

The primary objective was to describe how medical students use SoMe to learn about medicine. Secondary objectives were to evaluate their use of these platforms for choosing a medical specialty, analyze the prevalence of smartphone addiction in this population, and describe their potential misuse of SoMe. SoMe misuse was defined as the disclosure of information about hospital internships (text, photo, or video) that may breach patient confidentiality and the active internet-based search for private information [20].

### Ethical Considerations

The study was approved by the Ethics and Evaluation Committee for Non-Interventional Research of Rouen University Hospital (E2023-06). Participation was entirely voluntary. Participants were informed about the study's objectives and provided their consent before completing the survey. The survey was conducted anonymously, and no identifying information was collected or attempted to be gathered at any stage, ensuring participants’ privacy and confidentiality. No compensation was offered to participants, and they had the right to withdraw from the survey at any time without any consequences. According to institutional guidelines, as this was a noninterventional, anonymous survey with no personal health data collected, the study did not require further ethical exemptions or waivers beyond the initial approval.

### Population Selection

We conducted a prospective study in France using a declarative survey. The link to an open Google Form internet survey, consisting of 32 items on 1 web page, was emailed to the board of the French medical students’ association (Association des Etudiants en Médecine de France). This board forwarded the questionnaire to the association’s representatives at each of the 35 medical schools in France. These 35 representatives were instructed to share the link with their respective faculty’s students via email. All contacted students were asked to forward the survey link to their colleagues. All participants received information about the survey objectives, which were reiterated in the questionnaire’s introduction. There was no incentive to answer the questionnaire. As responses were anonymous, no information was collected to prevent multiple entries by the same individual. Also, as the questionnaire was open-ended, anyone with the link could answer it. The distribution via medical student representatives was intended to restrict the questionnaire’s visibility to the target audience only.

This survey was developed according to available guidelines for self-administered surveys [21]. Responses were submitted on a single web page with 1 “submit” button, which only allowed submissions via these unique links, making uninvited responses extremely unlikely. The request was sent to 35 representatives in France; however, as we were unable to determine how many students the request was forwarded to, we do not know the overall number of students who received the request to participate in the survey. We did not organize any specific follow-up on the distribution of the questionnaire with the contacted representatives. The survey was conducted in accordance with the Checklist for Reporting Results of Internet E-Surveys (CHERRIES; Checklist 1) [22].

The participants included in the analysis were medical students in their first (second to third year) and second (fourth to sixth year) cycles of medical study. The medical curriculum in France consists of 6 years of study before residency, although the first year of medical school is a competitive year with a success rate of about 15%; students in this year of study were not included in this analysis, which concerned only students who were certain to become physicians after their studies.

### Survey Design

The survey was developed by a team consisting of medical students, medical educators, and an expert in SoMe to ensure comprehensive coverage of relevant topics. The questions in the “use of social network” sections were designed based on a thorough review of existing literature on SoMe usage in medical education and consultations with subject matter experts [1,3,10]. The initial draft was reviewed by a panel of medical educators and students to assess face validity and to validate the list of social networks that met our definition. To further ensure the validity and reliability of the survey, a pilot test was conducted with the participation of the board of the French Medical Students’ Association to ensure the comprehensibility and relevance of the questions. Feedback from the pilot test was used to refine the survey questions for clarity and relevance. The psychometric properties of the survey were not formally assessed.

The survey consisted of 3 sections. The “demographic data” section collected information on city, age, gender, year of study, and whether the student had already retaken an exam (catch-up exam). The “use of social networks” section gathered data on personal and professional use of WhatsApp, types of social networks consulted at least once a week, average daily time spent on SoMe, usage of SoMe for learning about medicine and choosing a medical specialty, and the misuse of SoMe (searching for a patient’s name and spreading information from hospital internships).

We defined the sharing of content from hospital internships on SoMe as a misuse based on professional standards. It is generally considered unprofessional for students to share details about their clinical experiences on SoMe, as this behavior can undermine patient confidentiality and trust. The Health Insurance Portability and Accountability Act (HIPAA) regulations in the United States clearly state that sharing any patient information on SoMe is unacceptable [23] and medical societies have issued strict recommendations against such practices [24]. Since most student internships involve direct patient contact, sharing content related to these internships often involves discussing patient care experiences, which is inappropriate even if no identifiable patient information is shared.

Students also rated, using a 6-point Likert scale (1 point=“completely irrelevant” and 6 points=“completely relevant”), whether they thought it was appropriate to offer a teaching module on the professional or educational use of SoMe in medical school.

Finally, the “assessment of smartphone addiction” section evaluated smartphone addiction using the short version of the Smartphone Addiction Scale Short-Version (SAS-SV) developed by Kwon et al [25]. The SAS-SV was already translated into French, demonstrating the validity and reliability of the translated version adapted to French [26]. The scale comprises 10 positive 6-point Likert questions describing smartphone usage, with the total score ranging from 10 to 60 and higher scores indicating a greater risk of addiction. According to the threshold recommended for student populations, and given previous data concerning the absence of gender difference for the cutoff value of the SAS-SV among French-speaking students, the cutoff point was determined as superior or equal to 32 points to identify smartphone addiction [25,26]. The SAS-SV scale covers 6 addictive symptoms and they are loss of control, disruption of family or schooling, disregard for consequences, withdrawal, preoccupation, and tolerance. Each item is associated with an addictive symptom, except for 4 item clusters that are items 1 and 8 (both assessing “loss of control”), items 2 and 10 (“disruptions”), items 3 and 7 (“disregard for consequences”), and items 4 and 5 (“withdrawal”) [26]. As previously described, a rating of 4 or higher for each symptom was considered to signify the presence of this specific symptom [26]. Participants had to answer all questions to validate the questionnaire but could go back at any time to change their answers before the final validation. The original version of the questionnaire and its English translation can be found in Multimedia Appendices 1 and 2.

### Statistical Analysis

The values are presented as the number and percentage (n, %) for qualitative variables and as the median (IQR) for quantitative variables. Statistical analyses were performed in complete case analysis on fully completed questionnaires [27]. After ensuring the abnormal distribution of the data via a Shapiro-Wilk test, quantitative variables were compared using a Mann-Whitney test. Qualitative variables were analyzed using a Fischer exact test. The Spearman correlation test was used to assess the strength of the association between 2 quantitative variables. All statistical tests were 2-sided, and the *P*<.05 probability threshold was used to establish statistical significance. All statistical analyses were performed using R (version 4.1.3; R Core Team).

## Results

### Demographic Data

The compilation of responses took place from May 22 to October 26, 2021. A total of 762 medical students responded to the survey. Among them, the median age was 22 (IQR 21‐24) years, and the gender ratio was 0.39 (212 males and 547 females; 3 students identified themselves as “gender neutral/non-gendered”). Respondents came from all the French metropolitan regions (Table 1). The participants were distributed as 149 (19.5%) second-year students, 121 (15.9%) third-year students, 139 (18.2%) fourth-year students, 119 (15.6%) fifth-year students, and 234 (30.7%) sixth-year students. Among the 762 respondents, 287 (37.7%) had retaken an exam at least once during their medical curriculum.

**Table 1.** Distribution of respondents by region.

Region	Respondents, n (%)
Auvergne-Rhône-Alpes	105 (13.8)
Bourgogne-Franche-Comté	106 (13.9)
Bretagne	21 (2.8)
Centre-Val de Loire	29 (3.8)
Grand Est	25 (3.3)
Hauts-de-France	24 (3.2)
Normandie	134 (17.6)
Nouvelle-Aquitaine	24 (3.2)
Occitanie	88 (11.5)
Pays de la Loire	61 (8.0)
Provence-Alpes-Côte d’Azur	90 (11.8)
Île-de-France	55 (7.2)

### Use of SoMe

Among the 762 respondents included, 624 (81.8%) were WhatsApp users and 762 (100%) were SoMe users, spending a median time of 120 (IQR 60‐150) minutes per day on them. A total of 555 (72.8%) students felt that their time spent on SoMe impacted their study time and 656 (86.1%) used SoMe to learn about medicine. The 3 most used SoMe for this purpose were YouTube (504/762, 66.1%), Instagram (433/762, 56.8%), and Facebook (320/762, 42%; Table 2). A total of 115 (15.1%) students used WhatsApp for professional purposes (internship questions, exchange of night shifts, and discussion about a patient).

**Table 2.** Proportion of medical students using specific social networks for medical education, exploration of medical specialties prior to selection, and sharing content related to hospital internship.

SoMe^a^	Usage, n (%)		
	Exploring medical specialties	Medical education	Sharing content about hospital internships
Facebook	235 (30.8)	320 (42.0)	47 (6.2)
Instagram	375 (49.2)	433 (56.8)	157 (20.6)
LinkedIn	2 (0.3)	3 (0.4)	1 (0.1)
Pinterest	1 (0.1)	7 (0.9)	0 (0)
Reddit	3 (0.4)	8 (1.0)	0 (0)
Snapchat	2 (0.3)	9 (1.2)	68 (8.9)
TikTok	9 (1.2)	20 (2.6)	1 (0.1)
Twitch	1 (0.1)	0 (0)	0 (0)
Twitter	70 (9.2)	63 (8.3)	18 (2.4)
YouTube	220 (28.9)	504 (66.1)	0 (0)

a SoMe: social media.

On SoMe, 79.1% (604/762) students followed 1 or more physicians whom they knew (resident or senior physician), and 25.9% (197/762) followed 1 or more national medical societies. A total of 522/762 (67.9%) students used SoMe to learn about a medical specialty in anticipation of choosing one. We identified significant misuse of social networks in a professional context, as 27.2% (207/762) of students had already posted content on SoMe (text, photo, and video) related to their hospital internship, and 10.8% (82/762) had ever searched for a patient’s name on a SoMe platform. SoMe that used to post content related to hospital internships are presented in Table 2. Regarding the interest in teaching modules on the professional or educational use of SoMe in medical school, 61.4% (468/762) students found this proposal relevant (204/762, 26.7%), very relevant (156/762, 20.4%), or completely relevant (108/762, 14.2%). The remaining students (294/762, 38.6%) did not find this teaching relevant.

### Smartphone Addiction

**Figure 1.**
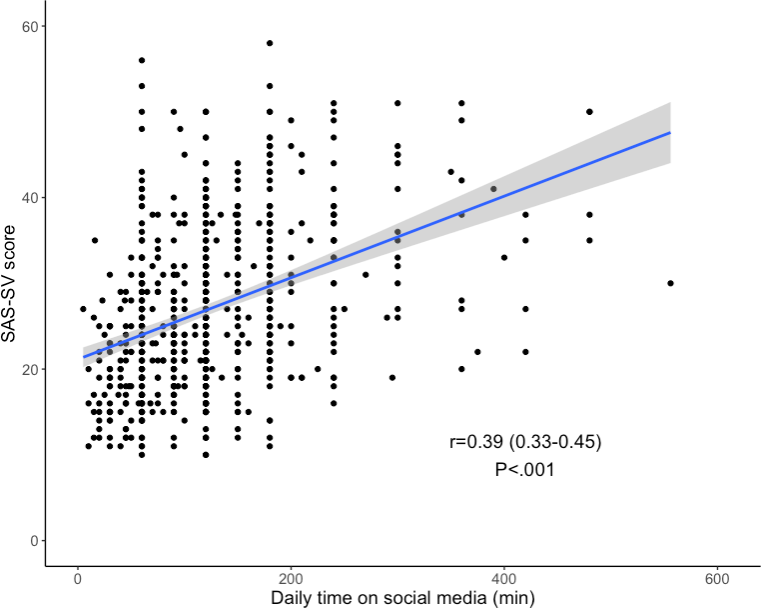
Correlation between the time spent on social media and the SAS-SV score. SAS-SV: Smartphone Addiction Scale Short-Version.

Among the 762 students analyzed, 222 (29.1%) had an SAS-SV score of at least 32/60, defining smartphone addiction. Nonaddicted students had a median SAS-SV score of 24 (IQR 20‐27), while addicted students had a median score of 37 (IQR 35‐42; *P*<.001). There was a significant correlation between the time spent on SoMe and the SAS-SV score (*r*S=0.39, 95% CI 0.33‐0.45; *P*<.001; Figure 1). There were no demographic differences between smartphone-addicted and nonaddicted students (Table 3). However, addicted students spent more time on SoMe, with a more frequent impact on their study time, and a higher tendency to post content from their hospital internships on SoMe (Table 3). Among the 222 addicted students, the most frequent addiction symptoms were tolerance (207/222, 93.2%), loss of control (166/222, 74.8%), disruption of family or schooling (138/222, 62.2%), and withdrawal (127/222, 57.2%). Only a few students displayed disregard for consequences symptoms (30/222, 13.5%) and none presented preoccupation about their smartphone use.

**Table 3.** Characteristics and social media behavior of medical students with or without smartphone addiction.

	Overall (n=762)	Nonaddicted students (n=540)	Addicted students (n=222)	*P* value
**Gender, n (%)**				.36
Male	212 (27.8)	150 (27.8)	62 (27.9)	
Female	547 (71.8)	389 (72.0)	158 (71.2)	
Other	3 (0.4)	1 (0.2)	2 (0.9)	
**Age (years), median (IQR)**	22 (21‐24)	22 (21‐24)	22 (21‐23)	.71
**Cycle of study, n (%)**				.91
First cycle	270 (35.4)	192 (35.7)	78 (35.1)	
Second cycle	492 (64.6)	348 (64.3)	144 (64.8)	
**Retook an exam, n (%)**				.09
Yes	287 (37.7)	193 (35.7)	94 (42.3)	
No	475 (62.3)	347 (64.3)	128 (57.7)	
**Time spent on social media (minutes per day), median (IQR)**	120 (60‐150)	94 (60‐120)	150 (120‐200)	<.001
**Impact on the study time, n (%)**				<.001
Yes	555 (72.8)	344 (63.6)	211 (95.0)	
No	207 (27.2)	196 (36.4)	11 (5.0)	
**Social media use to learn about medicine, n (%)**				.98
Yes	656 (86.1)	465 (86.0)	191 (86.0)	
No	106 (13.9)	75 (14.0)	31 (14.0)	
**Posts related to the hospital internship, n (%)**				.002
Yes	207 (27.2)	129 (23.8)	78 (35.1)	
No	555 (72.8)	411 (76.2)	144 (64.9)	
**Ever searched a patient’s name on social media, n (%)**				.07
Yes	82 (10.8)	51 (9.4)	31 (14.0)	
No	680 (89.2)	489 (90.6)	191 (86.0)	

### Subgroup Analyses

First- and second-cycle students show different patterns of social network use. Compared to their second-cycle peers, first-cycle students are less likely to use Facebook for their medical education (77/270, 28.5% vs 243/492, 49.3%; *P*<.001), and more inclined to use Instagram or Snapchat for example (Figure S1 in Multimedia Appendix 3). Smartphone addiction is the same between first- and second-cycle students (78/270, 28.9% vs 144/492, 29.3, respectively; *P*=.91), but graduate students are more likely to exhibit SoMe misuse behaviors (Table S1 in Multimedia Appendix 3).

Regarding gender differences, female and male students spent the same amount of time on SoMe (median 120, IQR 60‐150 minutes per day in both groups; *P*=.80) and had the same prevalence of smartphone addiction (158/547, 28.9% vs 62/212, 29.2% respectively; *P*=.92; Table S2 in Multimedia Appendix 3). They presented differences in the use of different social networks (Figure S2 in Multimedia Appendix 3). Female students were more likely to use SoMe for medical education (482/547, 88.1% vs 173/212, 81.6%; *P*=.04). The 2 genders showed different patterns of misuse, with a greater tendency to post content relating to hospital internships for female students (160/547, 29.1% vs 47/212, 21.8%; *P*=.04) and a greater tendency to search a patient’s name on SoMe for male students (33/212, 15.6% vs 49/547, 9%; *P*=.009).

## Discussion

### Principal Findings

This nationwide web-based survey aimed to describe the use of SoMe by medical students in France for their medical education and to evaluate the prevalence of smartphone addiction in this population. Our findings indicate that among the respondents, a significant majority (656/762, 86.1%) used SoMe to learn about medicine, with YouTube, Instagram, and Facebook being the most popular platforms. Respondents reported spending a substantial portion of their day on SoMe, with a median time of 120 (IQR 60‐150) minutes per day. However, misuse of SoMe was also reported, with 10.8% (82/762) of students searching for patients’ names on SoMe platforms. Nearly one-third (222/762, 29.1%) of respondents met the criteria for smartphone addiction according to the SAS-SV. Our study is the first nationwide survey to explore the use of SoMe for medical education among medical students in France and to investigate smartphone addiction on such a large scale in this specific population.

### Use of SoMe for Medical Education

Our study highlights the extensive use of SoMe among respondents for medical learning mainly by following physicians’ accounts (603/762, 79.1%) and, to a lesser extent, by following scientific societies (197/762, 25.9%). The main SoMe platforms used for this purpose were YouTube and Instagram, suggesting a preference for visual and multimedia content over textual information. While our survey did not assess whether SoMe were used more often than school-provided content, it indicates that SoMe was a significant supplementary source to traditional medical education, fostering collaboration among students and health care professionals. The COVID-19 pandemic has likely accelerated this trend [13,14], making it crucial for medical schools and educators to recognize the potential of these platforms and to integrate SoMe effectively into their teaching strategies.

However, it should be noted that 72.8% (555/762) of students surveyed felt that their time spent on SoMe negatively impacted their study time, highlighting the ambivalence of these platforms. Although students use SoMe for educational purposes, they also engage with these platforms for noneducational activities, which can detract from their study time. This point is acknowledged by the respondents, as 61.4% (468/762) of them found it relevant to add a teaching module on the professional or educational use of SoMe in medical school.

It is important to note that the quality and reliability of educational content on SoMe can vary widely [28,29]. There is also a need to consider how to produce and validate medical educational content on these platforms. Some authors proposed their personal guidelines on this topic, but there is still a lack of large consensus on how to provide medical education on SoMe [30,31]. Medical teachers who publish and moderate content on SoMe should probably be valorized at an institutional level, as they play a significant role in the current dissemination of knowledge to students. However, further research is still needed to evaluate the impact of these resources on students’ medical knowledge, skills, and professional development.

We describe that 79.1% (603/762) of students follow a physician they already know on SoMe and that 68.5% (522/762) use SoMe to help them choose a specialty. This is an important message, showing that physicians who are active on SoMe are seen (and are likely to be imitated) by students. This reinforces the absolute necessity to maintain perfect professionalism when communicating on SoMe as a health professional or as a teacher. Our results also suggest that SoMe could be an effective communication tool for medical academic societies to promote and present their specialty among students.

### Misuse of SoMe in a Professional Context

Our study also identified significant misuse of social networks in a professional context. Posting content related to hospital internship was reported by 27.2% (207/762) of respondents, and 10.8% (82/762) admitted to searching for patients’ names on SoMe platforms. Second-cycle students were more likely to present misuse with the risk of a breach of confidentiality, which is consistent with the fact that they spend more time on in-hospital internships than first-cycle students. We also observed a gender difference in misuse behavior, with female respondents more likely to post internship-related content and male respondents more likely to search for patient information. These results have already been reported in other studies [32,33]. These behaviors pose ethical and legal concerns, as they can lead to breaches of confidentiality and compromise patient privacy. Additionally, these actions may have severe consequences for medical students and their future careers, including disciplinary action and damage to their professional reputation. French law strictly forbids sharing any medical information with anyone other than the caring team, and any information apart from the ones strictly necessary to the patient’s course of treatment or care, except for some listed exceptions [34].

The platforms that seemed to be more problematic were Snapchat and Instagram, possibly due to the sensationalist nature of sharing photographs or videos, particularly ephemeral ones (“stories”). This is particularly worrying, as we recently showed that, on SoMe, photographs are much more likely to breach medical confidentiality than written posts [35]. In contrast, YouTube, which requires more effort to upload a video, appears much less likely to be used to share such content. Therefore, we suggest for the first time that the risk of SoMe misuse and medical confidentiality breaches seem to vary greatly among the different platforms. This information should probably be used to provide relevant information on the individual risks of each SoMe, which are not a uniform entity.

This problem of unprofessional use of SoMe by medical students is the subject of significant research literature, with which our results are consistent. Some authors report interesting results after implementing “social media and professionalism” course in medical school, with improvement in students’ SoMe behavior [36]. It is crucial for all medical schools and health care institutions to address these issues and promote the responsible use of SoMe among their medical students through the development of policies and educational interventions. SoMe misuse, particularly breaches of confidentiality, is not only a problem among students but also among physicians. Ahmed et al [32] identified tweets from 656 health care professionals’ Twitter profiles, including 486 (74.1%) doctors. Through these tweets, friends and family were able to identify clinical scenarios in 242 of the 754 (32.1%) tweets. In a study of the profiles of anesthesia and intensive care professionals, 5.3% of doctors’ accounts had posted content posing a confidentiality problem [35].

### Prevalence and Impact of Smartphone Addiction

Our findings indicate a concerning prevalence of smartphone addiction among medical students, with nearly one-third (222/762, 29.1%) of respondents meeting the criteria for addiction. This finding is consistent with the literature, which reports a smartphone addiction rate between 15% and 40% among students [19,37-39]. The SAS-SV score for addiction diagnosis is well established, and this scale has been already validated in French, increasing its reliability and reproducibility [26]. Our results showed a significant correlation between increased length of SoMe use and SAS-SV score. It is impossible to establish a causality link here, as students can become addicted to these platforms through overuse, but they may also use them intensively because of their addiction. In a Norwegian student population, Hjetland et al [40] also found a strong association between addiction and daily time spent on SoMe, particularly when this usage occurred in the evening.

The negative psychological, social, and physical effects of smartphone addiction are well described—lower academic performance, sleep disorders, anxiety, musculoskeletal disorders, severe social withdrawal, decreased physical activity, and hypertension [16-19]. It is thus recognized that this addiction has a negative impact at the individual level. However, our results also showed that addicted students reported more impact on their study time and a higher tendency to share hospital internship content on social networks, suggesting that students’ smartphone addiction could also have a negative impact on patients. There are no data on the potential link between medical students’ smartphone addiction and unprofessional behavior on SoMe, and further works are needed to explore this hypothesis.

### Limitations

Despite these interesting results, our work has several limitations. First, the response rate was low. Even if we were unable to determine the total number of medical students who received the survey invitation, the targeted population is approximately 42,000 students (which would give a response rate of 1.8% if we consider that all students received the invitation). As a result, it is not possible to estimate the nonresponse bias, which is probably significant, as students who chose to participate in the study might have different characteristics or behaviors than those who did not. Additionally, the study relied on self-reported data, which may be subject to recall and social desirability biases. Participants might have underreported their SoMe use or smartphone addiction due to concerns about stigma or privacy. Furthermore, the mode of distribution, an online survey distributed via email, may have introduced a selection bias. It is possible that students who are more active on SoMe were more likely to respond to the survey, potentially leading to an overestimation of the prevalence of SoMe use and smartphone addiction among the general population of medical students. Conversely, respondents may have different patterns of SoMe use compared to nonrespondents, which we were unable to assess.

Moreover, the response rate among female students (547/762, 71.8%) was significantly higher compared to male students. According to national statistics, the gender distribution in French medical schools was approximately 66% female and 34% male in 2021 [41]. This overrepresentation of female respondents might have influenced the results, as female students could have different SoMe usage patterns and concerns compared to their male counterparts. This limits the generalizability and interpretation of our findings to the overall population of medical students.

To address these limitations, future research should aim to achieve a higher and balanced response rate, possibly by using multiple distribution methods and follow-ups to reach a more representative sample of the student population. Additionally, qualitative studies could explore the reasons behind nonresponse and differences in SoMe patterns among different student demographics.

The use of the SAS-SV as the sole instrument to assess smartphone addiction has its limitations. While the SAS-SV has demonstrated good validity and reliability, it may not capture the full spectrum of addictive behaviors related to smartphone use. Further research could enable a better understanding of these behaviors in the medical student population and, more specifically, their impact on medical students’ results at the national final examination at the end of the sixth year of the medical course. Furthermore, the cross-sectional design of our study does not allow us to establish causal relationships between the use of social networks, smartphone addiction, and the potential consequences on medical students’ academic performance and well-being. Longitudinal studies would be required to better understand the directionality of these relationships. In addition, the psychometric properties of the “use of social network” section were not formally assessed, which is acknowledged as a limitation of this study. Future research should include a comprehensive psychometric evaluation to confirm the reliability and validity of the survey instrument.

### Conclusions

In conclusion, this study highlights the extensive use of SoMe for medical education among respondents and the concerning prevalence of smartphone addiction. Educators should recognize the potential of these platforms, promote responsible use, and address addiction issues. Further research is needed to optimize SoMe usage for medical education while minimizing risks associated with excessive use and addiction.

## Supplementary material

10.2196/55149 Multimedia Appendix 1 Original survey.

10.2196/55149 Multimedia Appendix 2 Web-based survey in English.

10.2196/55149 Multimedia Appendix 3 Secondary analyses.

10.2196/55149 Checklist 1 Reporting Results of Internet E-Surveys (CHERRIES) checklist.

## Abbreviations

CHERRIES: Checklist for Reporting Results of Internet E-Surveys
HIPAA: Health Insurance Portability and Accountability Act
SAS-SV: Smartphone Addiction Scale Short-Version
SoMe: social media
