# Supplementary material for: Social Media Usage for Medical Education and Smartphone Addiction Among Medical Students: National Web-Based Survey
Source: JMIR Med Educ. 2024 Oct 22;10:e55149. doi: 10.2196/55149 (PMC11526414; doi:10.2196/55149)
Supplement: Multimedia Appendix 1 [file mededu-v10-e55149-s001.docx]

L'objectif de cette étude est d'analyser les habitudes d'utilisation des principaux réseaux sociaux des étudiants en médecine français en ciblant particulièrement l'utilisation professionnelle ou à but d'enseignement de ces réseaux. Ce questionnaire est destiné aux étudiants de DFGSM2, DFGSM3, DFASM1, DFASM2 et DFASM3.
La durée de remplissage de ce questionnaire est d'environ 10 minutes.
Merci de votre participation et de votre aide pour cette étude.

**Données démographiques**

**Q1 : Quelle est votre faculté de médecine ?**

**Q2 : Quel est votre âge ? (En années)**

**Q3 : Quel est votre genre ? Homme / Femme / Autre – non genré**

**Q4 : Quelle est votre année d'étude ?**

- DFGSM 2
- DFGSM 3
- DFASM 1
- DFASM 2
- DFASM 3

**Q5 : Avez-vous déjà redoublé une année d'étude de médecine ?** Oui / Non

**Q6 : Avez-vous déjà été obligé de repasser un examen au rattrapage ?** Oui / Non

**Utilisation des réseaux sociaux**

**Q7 : Avez-vous l'application WhatsApp sur votre smartphone ?** Oui / Non

**Q8 : Si oui, utilisez-vous l'application WhatsApp dans un contexte professionnel avec d'autres étudiants (questions de stage, échange de gardes, discussion sur un patient, etc.) ?** Oui / Non

**Q9 : Parmi les réseaux sociaux et applications suivants, le- ou les-quel(s) utilisez-vous régulièrement (au moins une fois par semaine) ?**

- Facebook
- YouTube
- Instagram
- Snaphchat
- Twitter
- Pinterest
- TikTok
- Linkedin
- Twitch
- Reddit
- Je ne consulte pas (ou très peu) les réseaux sociaux

**Q10 : Si vous utilisez fréquemment les réseaux sociaux, combien de temps passez-vous dessus en moyenne par jour (en minutes) ?** [Pour information, il est possible de voir la durée d'utilisation d'une application sur la dernière semaine dans le menu batterie de votre smartphone]

**Q11 : Pensez-vous que votre temps de consultation des réseaux sociaux impacte votre temps passé à étudier ?** Oui / Non

**Q12 : Utilisez-vous du contenu trouvé sur des réseaux sociaux pour vous former à la médecine (films, fils d'enseignement, etc.) ?** Oui / Non

**Q13 : Si vous utilisez du contenu trouvé sur des réseaux sociaux pour vous former à la médecine, quel(s) réseau(x) social(aux) et/ou application(s) suivant(s) utilisez-vous ?**

- Facebook
- YouTube
- Instagram
- Snaphchat
- Twitter
- Pinterest
- TikTok
- Linkedin
- Twitch
- Reddit
- Je n'utilise pas les réseaux sociaux dans cet objectif

**Q14 : Suivez-vous un ou des médecin(s) (interne ou senior, que vous connaissez directement ou non) sur les réseaux sociaux ?** Oui / Non

**Q15 : Suivez-vous une ou des société(s) savante(s) médicale(s) sur les réseaux sociaux ?** [pour information, la liste des sociétés savantes médicales françaises est disponible ici: <https://fr.wikipedia.org/wiki/Liste_de_soci%C3%A9t%C3%A9s_savantes_scientifiques_en_France#Sciences_m%C3%A9dicales>] Oui / Non

**Q16 : Utilisez-vous les réseaux sociaux pour vous informer sur une spécialité médicale en vue d'un choix de spécialité d'internat ?** Oui / Non

**Q17 : Si vous utilisez les réseaux sociaux pour vous informer sur une spécialité médicale en vue d'un choix de spécialité d'internat, quel(s) réseau(x) social(aux) et/ou application(s) suivant(s) avez-vous consulté ?**

- Facebook
- YouTube
- Instagram
- Snaphchat
- Twitter
- Pinterest
- TikTok
- Linkedin
- Twitch
- Reddit
- Je n'utilise pas les réseaux sociaux dans cet objectif

**Q18 : Avez-vous déjà posté sur les réseaux sociaux du contenu (texte, photo, vidéo) en rapport avec vos stages hospitaliers ?** Oui / Non

**Q19 : Si vous avez déjà posté sur les réseaux sociaux du contenu (texte, photo, vidéo) en rapport avec vos stages hospitaliers, sur quel(s) réseau(x) social(aux) et/ou application(s) suivant(s) l'avez-vous fait ?**

- Facebook
- YouTube
- Instagram
- Snaphchat
- Twitter
- Pinterest
- TikTok
- Linkedin
- Twitch
- Reddit
- Je ne poste pas de contenu professionnel sur les réseaux sociaux

**Q20 : Avez-vous déjà cherché le nom d'un patient dans un moteur de recherche (ex: Google) ?** Oui / Non

**Q21 : Avez-vous déjà cherché le nom d'un patient sur une plateforme de réseau social ?** Oui / Non

**Q22 : Pensez-vous qu'il serait pertinent de proposer un module d'enseignement sur l'utilisation professionnelle ou à but d'enseignement des réseaux sociaux lors des études de médecine ? Pour chaque proposition vous devrez indiquer votre réponse sur une échelle de 1 (pas du tout pertinent) à 6 (tout à fait pertinent).** Échelle de Likert 1- Pas du tout pertinent à 6- Tout à fait pertinent

**Évaluation du degré d'addiction au smartphone**

Les 10 questions suivantes ont pour but de dépister une éventuelle addiction au smartphone. Pour chaque proposition vous devrez indiquer votre réponse sur une échelle de 1 (pas du tout d'accord) à 6 (tout à fait d'accord).

**Q23 : J'utilise mon smartphone de telle manière à ce que cela entraîne un impact négatif sur ma productivité/mon travail.** Échelle de Likert : 1-Pas du tout d'accord à 6-Tout à fait d'accord

**Q24 : J'ai du mal à me concentrer en cours ou durant le travail à cause du smartphone.** Échelle de Likert : 1-Pas du tout d'accord à 6-Tout à fait d'accord

**Q25 : Je ressens de la douleur aux poignets ou à la nuque quand j'utilise mon smartphone**. Échelle de Likert : 1-Pas du tout d'accord à 6-Tout à fait d'accord

**Q26 : Je ne supporte pas le fait de ne pas avoir mon smartphone.** Échelle de Likert : 1-Pas du tout d'accord à 6-Tout à fait d'accord

**Q27 : Je ressens de l'impatience et de l'irritation lorsque je n'ai pas mon smartphone.** Échelle de Likert : 1-Pas du tout d'accord à 6-Tout à fait d'accord

**Q28 : Je suis préoccupé par l'utilisation de mon smartphone, même lorsque je ne l'utilise pas.** Échelle de Likert : 1-Pas du tout d'accord à 6-Tout à fait d'accord

**Q29 : Je n'arrêterai jamais d'utiliser mon smartphone, même si son utilisation entraîne des conséquences négatives importantes dans ma vie quotidienne.** Échelle de Likert : 1-Pas du tout d'accord à 6-Tout à fait d'accord

**Q30 : Je surveille en permanence mon smartphone de manière à ne manquer aucune conversation (par ex. sur Twitter ou Facebook).** Échelle de Likert : 1-Pas du tout d'accord à 6-Tout à fait d'accord

**Q31 : J'utilise mon smartphone plus longtemps que je ne l'avais prévu.** Échelle de Likert : 1-Pas du tout d'accord à 6-Tout à fait d'accord

**Q32 : Mes proches me disent que j'utilise trop mon smartphone.** Échelle de Likert : 1-Pas du tout d'accord à 6-Tout à fait d'accord
