# Supplementary material for: Social Media Usage for Medical Education and Smartphone Addiction Among Medical Students: National Web-Based Survey
Source: JMIR Med Educ. 2024 Oct 22;10:e55149. doi: 10.2196/55149 (PMC11526414; doi:10.2196/55149)
Supplement: Multimedia Appendix 2 [file mededu-v10-e55149-s002.docx]

The aim of this study is to analyze the habits of French medical students on the main social networks, with a particular focus on the professional or educational use of these networks. This questionnaire is intended for DFGSM2, DFGSM3, DFASM1, DFASM2 and DFASM3 students.

This questionnaire takes approximately 10 minutes to complete.

Thank you for your participation and assistance in this study.

**Demographics**

**Q1: What is your medical school?**

**Q2: How old are you? (In years)**

**Q3: What is your gender? *** Man / Woman / Other - Non-gender

**Q4: What is your study year?**

- DFGSM 2 (2^nd^ year)
- DFGSM 3 (3^rd^ year)
- DFASM 1 (4^th^ year)
- DFASM 2 (5^th^ year)
- DFASM 3 (6^th^ year)

**Q5: Have you ever had to retake a year of medical school?** Yes / No

**Q6: Have you ever had to retake an exam?** Yes / No

**Use of social media**

**Q7: Do you have WhatsApp app on your smartphone?** Yes / No

**Q8: If so, do you use the WhatsApp application in a professional context with other students (internship questions, duty dates exchanges, discussions about patients, etc.)?** Yes / No

**Q9: Which of the following social networks and applications do you use regularly (at least once a week)?**

- Facebook
- YouTube
- Instagram
- Snaphchat
- Twitter
- Pinterest
- TikTok
- Linkedin
- Twitch
- Reddit
- I do not visit (or very few) social networks

**Q10: Do you think that your time visiting social networks impacts your time spent studying?** Yes / No

**Q11: If you use social networks frequently, how much time do you spend on it on average per day (in minutes)? [for information, it is possible to see the last weeks’ duration of use of an application in the battery menu of your smartphone]**

**Q12: Do you use content found on social networks to educate yourself about medicine (films, teaching threads, etc.)?** Yes **/** No

**Q13: If you use content found on social networks to educate yourself in medicine, which social network(s) and/or application(s) do you use?**

- Facebook
- YouTube
- Instagram
- Snaphchat
- Twitter
- Pinterest
- TikTok
- Linkedin
- Twitch
- Reddit
- I do not use social networks for this purpose

**Q14: Do you follow at least one physician (resident or MD, whom you know directly or not) on social networks?** Yes / No

**Q15: Do you follow one or more medical society(s) on social networks? [for information, the list of French medical societies is available here:** <https://en.wikipedia.org/wiki/Liste_de_soci%C3%A9t%C3%A9s_savantes_scientifiques_en_France#Sciences_m%C3%A9dicales>**]** Yes / No

**Q16: Do you use social networks to get information about a medical specialty for your choice of residency specialty?** Yes / No

**Q17: If you use social networks to get information about a medical specialty for your choice of residency specialty, which social network(s) and/or application(s) do you visit?**

- Facebook
- YouTube
- Instagram
- Snaphchat
- Twitter
- Pinterest
- TikTok
- Linkedin
- Twitch
- Reddit
- I do not use social networks for this purpose

**Q18: Have you already posted content on social networks (text, photo, video) related to your hospital internships?** Yes / No

**Q19: If you have already posted content on social networks (text, photo, video) related to your hospital internships, on which of the following social network(s) and/or application(s) did you do so?**

- Facebook
- YouTube
- Instagram
- Snaphchat
- Twitter
- Pinterest
- TikTok
- Linkedin
- Twitch
- Reddit
- I don’t post professional content on social networks

**Q20: Have you ever searched for a patient’s name in a search engine (e.g., Google)?** Yes / No

**Q21: Have you ever searched for a patient’s name on a social networking platform?** Yes **/** No

**Q22: Do you think it would be relevant to offer a teaching module on the professional use of social networks during medical studies? For each proposal, you will need to indicate your answer on a scale from 1 (not at all relevant) to 6 (entirely relevant).**

Not at all relevant

- 1
- 2
- 3
- 4
- 5
- 6

Entirely relevant

**Assessment of the degree of smartphone addiction**

The following 10 questions are intended to detect a possible smartphone addiction. For each proposition you must indicate your answer on a scale of 1 (strongly disagree) to 6 (strongly agree).

**Q23: I use my smartphone in such a way that it has a negative impact on my productivity/work** (Likert scale: 1-Strongly disagree to 6-Strongly agree)

**Q24:** **I have trouble concentrating in class or during work because of the smartphone** (Likert scale: 1-Strongly disagree to 6-Strongly agree)

**Q25: I feel pain in my wrists or neck when using my smartphone** (Likert scale: 1-Strongly disagree to 6-Strongly agree)

**Q26:** **I can’t stand not having my smartphone** (Likert scale: 1-Strongly disagree to 6-Strongly agree)

**Q27:** **I feel impatience and irritation when I don’t have my smartphone** (Likert scale: 1-Strongly disagree to 6-Strongly agree)

**Q28:** **I’m concerned about using my smartphone even when I’m not using it** (Likert scale: 1-Strongly disagree to 6-Strongly agree)

**Q29: I will never stop using my smartphone, even if its use has significant negative consequences in my daily life** (Likert scale: 1-Strongly disagree to 6-Strongly agree)

**Q30: I constantly monitor my smartphone so that I don’t miss any conversations (e.g. on Twitter or Facebook)** (Likert scale: 1-Strongly disagree to 6-Strongly agree)

**Q31: I use my smartphone longer than I expected** (Likert scale: 1-Strongly disagree to 6-Strongly agree)

**Q32: People tell me I use my smartphone too much** (Likert scale: 1-Strongly disagree to 6-Strongly agree)
