# Supplementary material for: Social Media Usage for Medical Education and Smartphone Addiction Among Medical Students: National Web-Based Survey
Source: JMIR Med Educ. 2024 Oct 22;10:e55149. doi: 10.2196/55149 (PMC11526414; doi:10.2196/55149)
Supplement: Multimedia Appendix 3 [file mededu-v10-e55149-s003.docx]

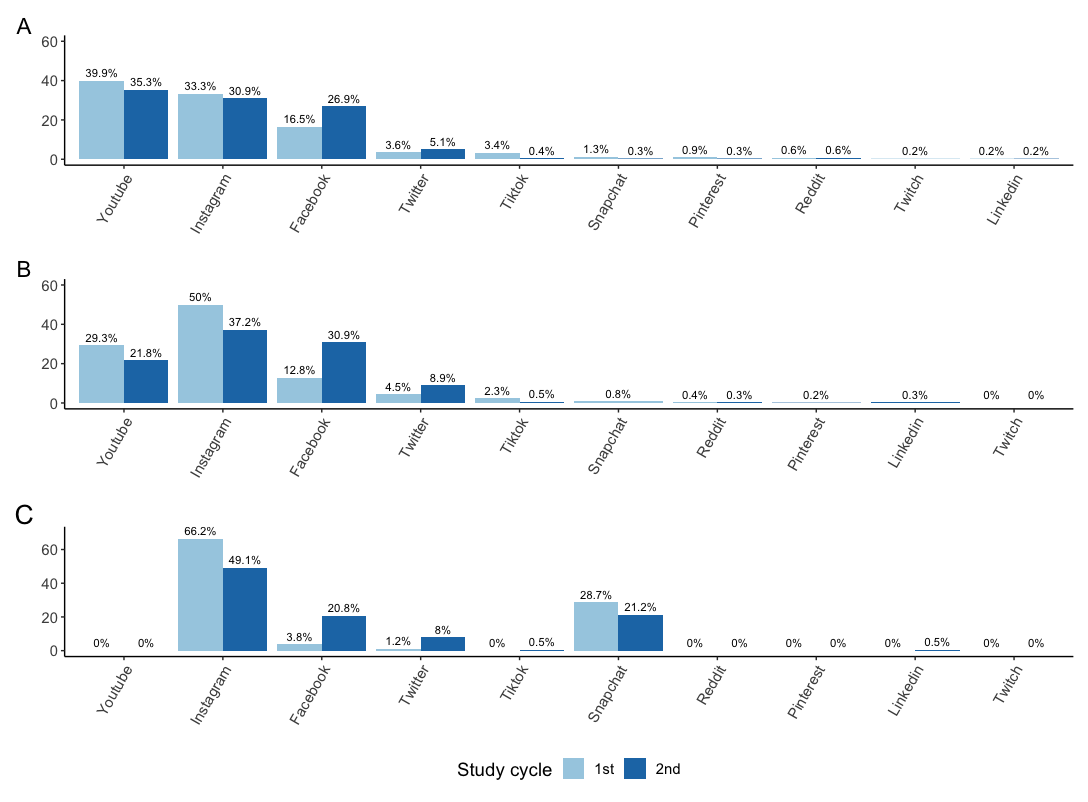


**Figure S1.** Proportion of medical students utilizing specific social networks for medical education among first and second cycles of medical studies (A), exploration of medical specialties prior to selection (B), and sharing content related to hospital internship (C).

**Table S1.** Characteristics and social networking behavior of first- and second-cycle medical students.

Data are presented as absolute values and percentages or median (IQR).

|  | **Overall**  **(N=762)** | **First cycle (n=270)** | **Second cycle (n=492)** | ***P*** |
| --- | --- | --- | --- | --- |
| **Gender**, n (%)   - **Male** - **Female** - **Other** | 212 (27.8)  547 (71.8)  3 (0.4) | 68 (25.2)  202 (74.8)  0 (0) | 144 (29.3)  345 (70.1)  3 (0.6) | .2 |
| **Age (years)**, median (IQR) | 22 (21-24) | 20 (20-21) | 23 (22-24) | <.01 |
| **Retook of an exam**, n (%) | 287 (37.7) | 57 (21.1) | 230 (46.7) | <.01 |
| **Time spent on social media (min/day)**, median (IQR) | 120 (60-150) | 120 (80, 180) | 120 (60, 150) | <.01 |
| **Impact on the study time**, n (%) | 555 (72.8) | 194 (71.9) | 361 (73.4) | .7 |
| **Social media use to learn about medicine**, n (%) | 656 (86.1) | 228 (84.4) | 428 (87.0) | .3 |
| **Post(s) related to the hospital internship**, n (%) | 207 (27.2) | 59 (21.9) | 148 (30.1) | .02 |
| **Ever searched a patient's name on social media**, n (%) | 82 (10.8) | 15 (5.6) | 67 (13.6) | <.01 |
| **Smartphone addiction**, n (%) | 222 (29.1) | 78 (28.9) | 144 (29.3) | >.9 |


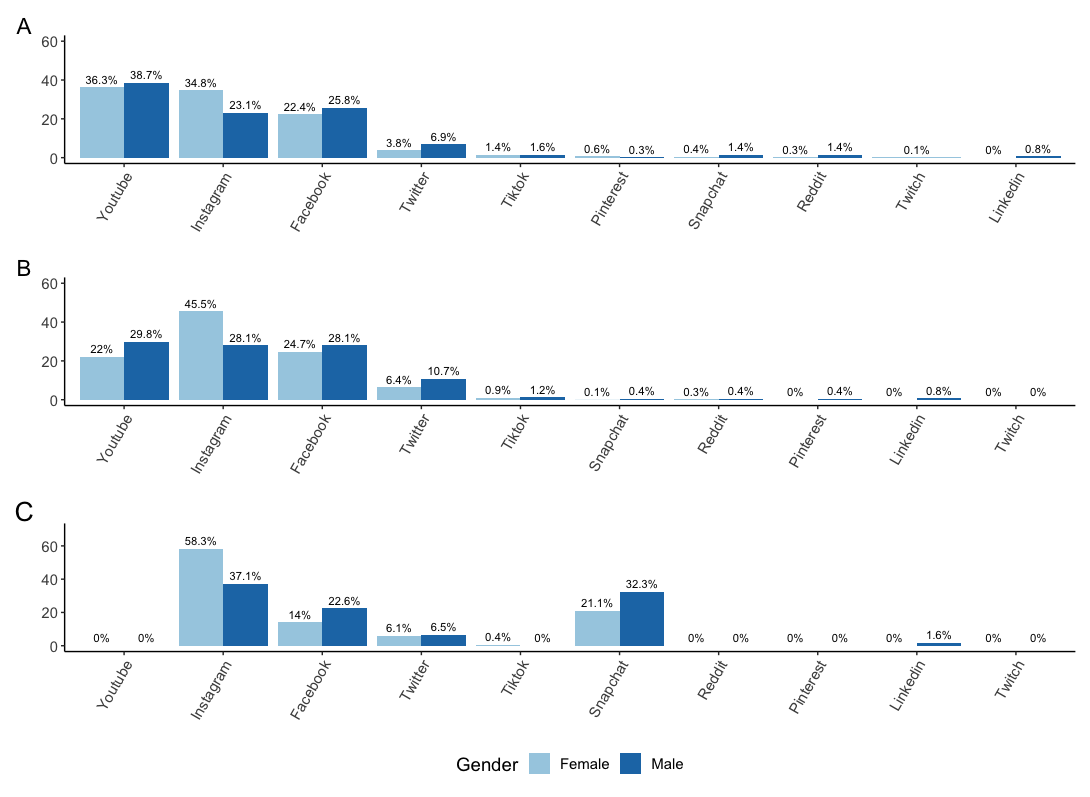


**Figure S2.** Proportion of medical students utilizing specific social networks for medical education among male and female students (A), exploration of medical specialties prior to selection (B), and sharing content related to hospital internship (C).

**Table S2.** Characteristics and social networking behavior of male and female students.

Data are presented as absolute values and percentages or median (IQR).

|  | **Overall**  **(N=759)** | **Female**  **(n=547)** | **Male**  **(n=212)** | ***P*** |
| --- | --- | --- | --- | --- |
| **Age (years)**, median (IQR) | 22 (21-24) | 22 (21-24) | 22 (21-24) | .3 |
| **Cycle of study**, n (%)   - **1^st^ cycle** - **2^nd^ cycle** | 270 (35.6)  489 (64.4) | 202 (36.9)  345 (63.1) | 68 (32.1)  144 (67.9) | .2 |
| **Retook of an exam**, n (%) | 285 (37.5) | 196 (35.8) | 89 (42.0) | .12 |
| **Time spent on social media (min/day)**, median (IQR) | 120 (60-150) | 120 (60-150) | 120 (60-150) | .8 |
| **Impact on the study time**, n (%) | 552 (72.7) | 408 (74.6) | 144 (67.9) | .06 |
| **Social media use to learn about medicine**, n (%) | 655 (86.3) | 482 (88.1) | 173 (81.6) | .04 |
| **Post(s) related to the hospital internship**, n (%) | 207 (27.2) | 160 (29.1) | 47 (21.8) | .04 |
| **Ever searched a patient's name on social media**, n (%) | 82 (10.9) | 49 (9.0) | 33 (15.6) | <.01 |
| **Smartphone addiction**, n (%) | 220 (29.0) | 158 (28.9) | 62 (29.2) | >.9 |
